# Indications Afforded by the State of the Iris in Cases of Cerebral Lesion

**Published:** 1843-08

**Authors:** 


*Indications afforded by the state of the Iris in cases of Cerebral Lesion.*—In the examination of cases of injury to the brain it is extremely important for you to enter minutely into all those signs which indicate any injury to the brain. First, the mental condition; next, the state of the pupils—the iris is placed before that expanded surface of the optic nerve, the retina, as an intelligent curtain to guard it from injury. The vital contrivances by which it acts, and by which its action is directed, are so beautifully perfect that the extent of the opening of the curtain is indicative of the state of the nervous apparatus it is destined to protect, by preventing such an amount of light impinging upon it as would be liable to injure it. In disease of the globe of the eye the dilated pupil indicates more or less pressure on the retina by some cause in the globe itself, such as a permanently turgid choroid, &c.

But if with a healthy eye, in connection with a blow on the head, we find a dilated pupil, then we have the sign of some pressure or injury to the nerve in its course within the skull, or the ganglia in which it terminates.

The dilated pupil, then, indicates very serious injury to the optic nerve, or the nervous centres with which it is connected, though it may happen, as in the case of very severe concussion, the injury is remediable. The contracted pupil, on the contrary, indicates an irritability of the nervous instruments, and undue excitement of their natural function—not an obliteration of it. You will sometimes see, in the case of injury of the brain, dilatation of one pupil and contraction of the other; where this is the case you will find the most severe injury of the brain on the side opposite the dilated pupil. I have several facts to prove this assertion, which I shall relate on a future occasion.—*Medical Gazette, Mr. Solly's Clinical Lecture: Medical Examiner.*

